# Comparative analyses across cattle genders and breeds reveal the pitfalls caused by false positive and lineage-differential copy number variations

**DOI:** 10.1038/srep29219

**Published:** 2016-07-06

**Authors:** Yang Zhou, Yuri T. Utsunomiya, Lingyang Xu, El Hamidi abdel Hay, Derek M. Bickhart, Tad S. Sonstegard, Curtis P. Van Tassell, Jose Fernando Garcia, George E. Liu

**Affiliations:** 1 Animal Genomics and Improvement Laboratory, BARC, USDA-ARS, Beltsville, Maryland 20705, USA; 2 College of Animal Science and Technology, Northwest A&F University, Shaanxi Key Laboratory of Agricultural Molecular Biology, Yangling, Shaanxi 712100, China; 3 Departamento de Medicina Veterinária Preventiva e Reprodução Animal, Faculdade de Ciências Agrárias e Veterinárias, UNESP—Univ Estadual Paulista, Jaboticabal, São Paulo, 14884-900, Brazil; 4 Institute of Animal Science, Chinese Academy of Agricultural Science, Beijing, 100193, China; 5 Departamento de Apoio, Produção e Saúde Animal, Faculdade de Medicina Veterinária de Araçatuba, UNESP—Univ Estadual Paulista, Araçatuba, São Paulo, 16050-680, Brazil

## Abstract

We compared CNV region (CNVR) results derived from 1,682 Nellore cattle with equivalent results derived from our previous analysis of Bovine HapMap samples. By comparing CNV segment frequencies between different genders and groups, we identified 9 frequent, false positive CNVRs with a total length of 0.8 Mbp that were likely caused by assembly errors. Although there was a paucity of lineage specific events, we did find one 54 kb deletion on chr5 significantly enriched in Nellore cattle. A few highly frequent CNVRs present in both datasets were detected within genomic regions containing olfactory receptor, ATP-binding cassette, and major histocompatibility complex genes. We further evaluated their impacts on downstream bioinformatics and CNV association analyses. Our results revealed pitfalls caused by false positive and lineage-differential copy number variations and will increase the accuracy of future CNV studies in both taurine and indicine cattle.

Copy number variation (CNV) is defined as deletions, insertions, and duplications ranging from 50 base pairs (bp) to 5 million base pairs (Mbp)^1^. This type of variation has drawn wide attention for its high diversity among different species, breeds and individuals, and for its drastic effects on gene expression and function, such as altering gene dosage, disrupting coding sequence or perturbing long-range gene regulation^2^. To date, CNV has been studied extensively in humans^1^,^3^,^4^,^5^,^6^,^7^, mice^8^,^9^,^10^ and domesticated animals^11^,^12^,^13^,^14^,^15^,^16^,^17^,^18^,^19^,^20^. In cattle, we first reported germline and somatic CNV in Holstein breed using array comparative genomic hybridization (CGH)^21^. We then analyzed the diversity of CNVs among 17 cattle breeds using a high density array CGH platform^19^. Short after, numerous studies about cattle CNV appeared focusing on single breeds such as Angus, Holstein, Hanwoo, Brown Swiss, Simmental and Qinchuan cattle^22^,^23^,^24^,^25^,^26^,^27^,^28^. Nellore cattle is a major *Bos indicus* breed and has been used in the cattle diversity studies; however, previous CNV studies mainly focused on CNV diversity in *Bos taurus*, and little attention has been given to lineage-differential CNVs between two subspecies: *Bos indicus* and *Bos taurus*. Additionally, the potential effects of misassembly of the taurine reference genome on the CNV detection has not been studied in array-based CNV surveys.

Several approaches have been used to identify CNVs, which included CGH array, SNP array and next-generation sequencing (NGS). No matter which method is used, the accuracy of the reference genome assembly influences both screening for novel mutations and genotyping for known mutations. For species with large and complex genomes, human has the best reference assembly as compared to others. Many other reference assemblies are still in the incomplete draft status, and often contain errors/misassemblies and gaps^29^. These reference assemblies also often only contain a haploid genome consisting of selected alleles derived from one or a small number of selected individual(s), thus not representing the combined DNA sequence of the whole species. In genetic variation analysis, these limitations (errors and gaps) in the reference genome can lead to false positive (FP) and false negative variant calls, respectively. One early study in human found that a total of 38.9 Mbp sequence was likely involved in assembly misassignment errors and identified a significant subset of SNPs that were potential paralogous sequence variants^30^. Another team recently identified assembly errors caused by false tandem duplications in chicken^31^. The cattle UMD3.1 assembly covers 29 autosomes and the X chromosome and contains many gaps and sequences on unplaced scaffolds^32^. Although UMD3.1 has no chrY, it does contain sequence reads from both the cow (female, L1 Dominette 01449) and its sire (male, L1 Domino 99375). Most of chrY sequences are found on unplaced scaffolds but occasionally some of them could be misplaced on autosomes or chrX. These misassignment errors would cause distorted dosage ratios when comparing between males/bulls and females/cows, thus led to false positive CNVs. This problem is even worse when variation analysis produces large data sets that cannot be individually validated or there is no independent cross-reference dataset or filter^33^,^34^.

On the other side, segmental duplications are well known events which may cause genome assembly errors and complicate conventional genomics studies^29^. CNV discovery studies often produce large calling datasets with certain part of them being false positive. Therefore, identifying misassembled regions in the reference genome will help to improve CNV calling and facilitate its downstream analyses. In this study, we detected CNVs based on large number of Nellore cattle using the BovineHD SNP microarray data. Due to the variable frequencies among the different parts within one CNVR, we divided the CNVRs to CNV segments and compared CNV segment frequencies between different genders and breeds. When comparing across genders, we found and independently validated 9 false positive CNVRs with a total length of 0.8Mb that were caused by incorrect scaffolding of sequence from chrX or chrY to the autosomes. We further evaluated the potential effects of these false positive CNVRs on the association study using two typical body traits. When comparing across cattle breeds, we also found one CNVR on chr5 showed high deletion frequency in Nellore cattle, which should be carefully considered when performing bioinformatics and association analyses in Nellore cattle.

## Results

### Genome wide CNV detection in Nellore cattle using PennCNV

We performed CNV calling on the UMD3.1 assembly using PennCNV in 2,230 Nellore cattle. After quality control filtering, 1,682 animals were kept for subsequent analyses. A total of 110,521 CNV events were detected, representing 15,457 distinct CNVs with an average length of 67,263 bp (Table S1). These CNVs were merged into 4,562 nonredundant CNVRs with a total length of ~186 Mb, corresponding to ~7.47% of the autosomal genome sequence (Table S2). This percentage was higher than the previous result that we reported for 674 HapMap samples, where CNVRs covered around ~5.85% of the genome^35^. In previous studies, more CNV loci were predicted in indicine breeds than in European taurine breeds, which is consistent with the known breed divergence and history^19^. In humans, the CNVR coverage has increased to 77.97% of the whole genome as the human CNV studies accumulated (http://dgv.tcag.ca/dgv/app/statistics). Our observation may also be related to the larger number of samples we used for this study than previous studies. Our PCR-based validation results showed around 70% of the 72 qPCR were consistent with the PennCNV predictions (Table S3). We detected 1,783 genes that overlapped with CNVRs and performed Gene Ontology analysis which showed consistent results with previous studies that immunity and sensory response-related genes are overrepresented^19^ (Table S4).

### Distribution of CNVRs on autosomal chromosomes

**Figure 1:**
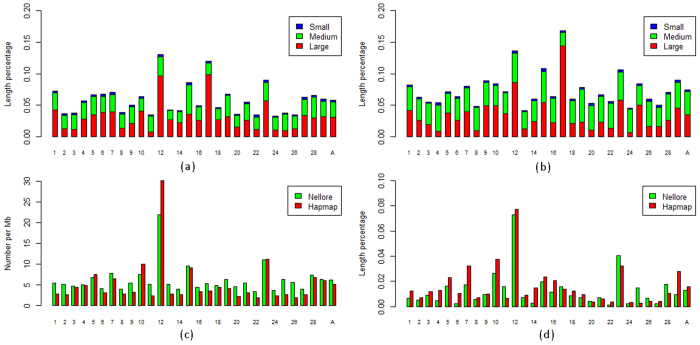
Comparisons of CNVR coverage between Nellore and HapMap cattle. Y-axis: the length percentage of CNVR coverage; X-axis: autosomes from chr1 to chr29 and A: average of all autosomes. (**a**) The coverage of CNVRs of different sizes on autosomes in Nellore cattle; CNVRs were assigned as large (length ≥ 100 kb); medium (10 kb ≤ length < 100 kb) or small (length ≤ 10 kb). (**b**) The coverage of CNVRs of different sizes on autosomes in HapMap cattle; (**c**) The counts of CNVs per Mbp in Nellore and HapMap cattle; (**d**) The coverage of CNVRs derived from CNV segments with a high frequency (>0.1) in HapMap cattle.

We calculated the combined CNVR coverage on each chromosome in Nellore cattle as compared to the Bovine HapMap data as reported in our previous study^35^ (Fig. 1a,b). The two results were roughly consistent in which CNVR coverage on chr12 or chr17 was near two times of the average and both their superiorities were mainly contributed by large CNVRs (length ≥ 100 kb). On most of other chromosomes, the medium length CNVRs (10 kb ≤ length < 100 kb) instead of the large CNVRs were the predominant contributors, especially on the chromosomes with low CNVR coverage (such as chr8, chr13 and chr24).

We used the number of CNVs per Mbp to evaluate the distribution of CNVs on different autosomes (Fig. 1c). The CNV count per Mbp on chr12 was the highest while that on chr17 was surprisingly lower than the genome-wide average. This chr17 result was probably related to the fact that its overlap regions among CNVs are also shorter than the average of autosomes (Figure S1). For chr12, we did not detect a significant overlap between genes and CNVRs when compared to other chromosomes. The percentage of genic regions on chr12 was lower than all other autosomes except for chr24 (Figure S2), and only 33 of the 1,783 genes that overlapped with Nellore cattle CNVRs were located on chr12. The high CNVR coverage on chr12 are in line with its high percentage of CNVRs within intergenic regions, where fewer functional genomic elements exist. Even though the overall negative correlation between CNVRs and gene distribution were not significant when we considered all autosomes, CNV events appeared near known, highly variable genes on some chromosomes. Such as chr23, where MHC genes were enriched, was ranked as the second in term of CNV count per Mbp. Additionally, chr15, where olfactory receptor (OR) genes were enriched, also had a high CNV count per Mbp.

Since over 80% of distinct CNVRs were merged from smaller and overlapping fragments, sub-regions of each CNVR will have different frequencies. We merged all distinct CNVs in both Nellore and HapMap cattle, divided them to CNV segments according to the boundaries of individual CNV calls^36^ (Table S5). CNV segments with frequencies lower than 0.01 took up over 70% of the total CNVR length. After filtering CNV segments with frequencies lower than 0.01, we constructed the coverage map of high frequency CNV segments for cattle autosomes (Fig. 1d). This result again confirmed chr12 as the chromosome with the highest CNVR coverage. A big decrease of CNVR coverage was found on chr17 also, which supported that CNVRs on chr17 were made of CNV segments with low frequency, probably caused by short overlaps between individual CNV calls.

### False positive CNVRs caused by misplacing sequence from sex chromosomes to autosomes

**Figure 2:**
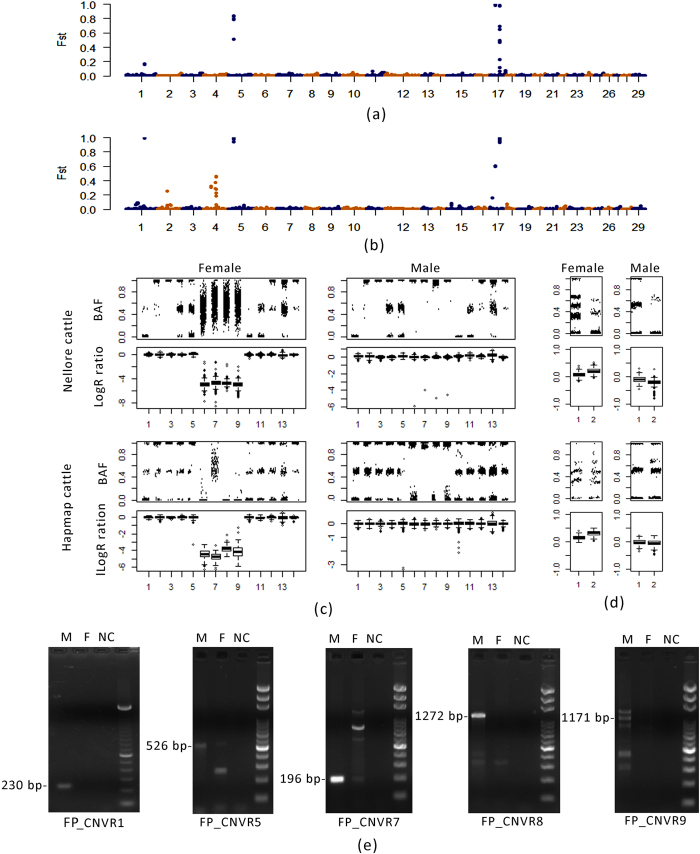
False positive CNVRs caused by cattle genome assembly errors. (**a**) *F*_*ST*_ between male and female of Nellore cattle at CNV segment level; (**b**) *F*_*ST*_ between male and female of HapMap cattle at CNV segment level; (**c**) BAF and logR ratio distribution of SNP probes near FP_CNVR7. Five upstream and downstream probes were included to represent the basal level; (**d**) BAF and logR ratio distribution of SNP probes within FP_CNVR2; (**e**) Male-specific PCR validation results. Although there were nonspecific bands in some samples, specific amplifications of target bands (labeled) were only present in males.

As mentioned earlier in Introduction, when sex chromosome sequence is incorrectly placed on autosomes, distorted dosage ratios between males/bulls and females/cows can cause false positive CNVs. Interestingly, some CNV segments did display distorted dosage ratios when we considered across animal genders. We compared the frequency differences between male and female cattle at global CNV level through F-statistics in either Nellore or HapMap cattle (Fig. 2a,b). Both populations showed over 99.7% CNV segments had *F*_*ST*_ value lower than 0.1 and some CNV segments were with abnormally high *F*_*ST*_ values on chr1, chr2, chr4, chr5 and chr17 (Table S6). Those CNV segments with *Fst* values higher than 0.9 in either Nellore or HapMap cattle were merged into 5 CNVRs with a total length of 0.67 Mbp. Because PennCNV combines LogR ratio (LRR) and B allele frequency (BAF) to predict CNVs^37^, we checked their distributions. All five CNVRs contained probes with low LRR and abnormal BAF patterns in female as compared to those in male samples (Fig. 2c; Figure S3). Additionally, there was a lack of heterozygote calls for male cattle in the probes that comprised the five CNVRs, which implied only one allele existed in each male sample. We further performed a similarity search using the sequence in the five CNVRs against chrY of the Btau_4.6.1 genome reference, which confirmed that the sequence of the five CNVRs belongs to the Y chromosome.

We also analyzed CNV segments with *F*_*ST*_ values higher than 0.15 on chr2, chr4 and chr17 either in Nellore or HapMap cattle. Contrary to the segments wrongly assembled from chrY to autosomes, all of these segments had higher gain copy number gain in females and low variant rates in males. The segments were merged into 4 CNVRs with 0.13 Mb. All average LRRs in the 4 CNVRs were near the basal level around 0 while they were slightly higher in female cattle than those in male cattle in both HapMap cattle and Nellore cattle (Fig. 2d; Figure S4). Moreover, abnormal distributions were seen in the BAF distribution, despite the fact that the probes clearly had clusters. The probes of FP_CNVR2 and FP_CNVR4 in female showed 5 clusters for BAF (at 0.0, 0.33, 0.5, 0.66, 1.0), which was more likely explained by two different loci (one with two alleles: 0.0, 0.5, 1.0; one with three alleles: 0.0, 0.33, 0.66, 1.0). We also searched the sequences of the four CNVRs against the Btau_4.6.1 genome reference. The sequence from FP_CNVR2 and FP_CNVR4 were found on unplaced scaffolds, and the sequence from FP_CNVR3 and FP_CNVR6 were found on chrX. These abnormal CNVRs were further confirmed by male-specific PCR and qPCR (Fig. 2e, Table S7). In total, we found around 0.8 Mbp CNVRs in length that may be caused by misassignment of sequence from sex-chromosomes to autosomes and labeled them as false positive (FP) CNVRs (Table 1).

### Differential CNVRs between Nellore and taurine cattle

We compared the Nellore data with the taurine data derived from the Bovine HapMap study to define the common and differential CNVRs between Nellore cattle with taurine breeds. First, we filtered the Nellore data in the following way: to minimize the effects of close relationships and genome sharing among samples, the highly related individuals with pi-hat (an identity-by-descent or IBD estimation) value over 0.4 were removed in the Nellore dataset^38^. After filtering, we had 882 Nellore cattle genotypes left for this comparison. For the taurine cattle, we excluded all African taurine and indicine-related samples in the HapMap data and the left 316 samples represented high diverse individuals of 12 European taurine breeds.

**Figure 3:**
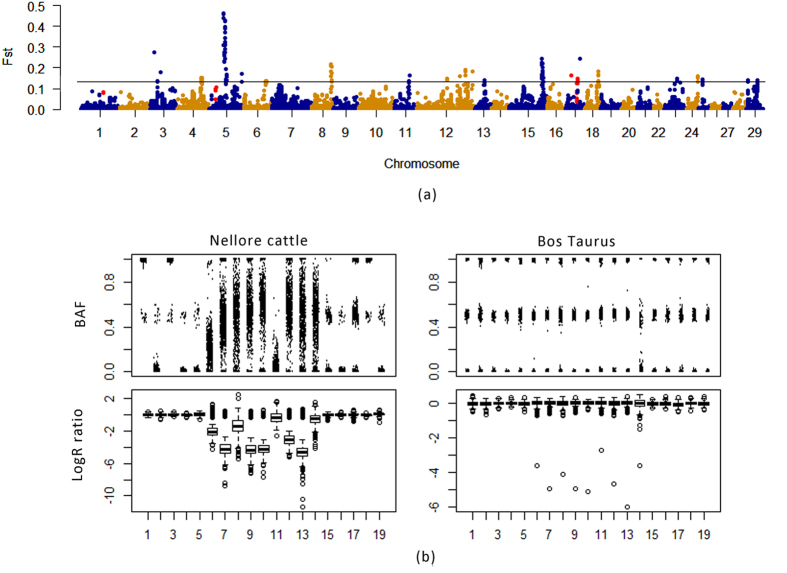
Comparisons of CNV segments between Nellore and taurine cattle. (**a**) *F*_*ST*_ between Nellore and taurine cattle at CNV segment level. The red dots represent the false positive CNV segments. (**b**) BAF and logR ratio distribution of SNP probes in Diff_CNVR7. Five upstream and downstream probes were included to represent the basal level.

Previous studies have used CNVRs merged across individual calls from different populations without accounting for their frequencies within each population sub-group. However, CNVRs are created by merging CNVs with different frequencies, which lead to discrete frequencies in different parts of one CNVR. We again merged all distinct CNVs in all selected Nellore and taurine samples, and partitioned CNVRs to CNV segments based on CNV boundaries. We observed a total of 13,016 CNV segments which also included the segments in the 0.8 Mbp false positive CNVRs (Table S8). Then we used F-statistics (cutoff threshold of *F*_*ST*_ ≥ 0.14, i.e. 0.5% tail) to evaluate the differentiation between Nellore and taurine cattle at CNV segment level (Fig. 3a; Table S8). We obtained 130 divergent CNV segments with 42 of them being enriched on chr5 (Table S9). The segments were merged into 36 CNVRs with total length of 1.22 Mbp; however, we found two CNVRs with 0.13 Mbp on chr17 were actually located in the false positive CNVRs and they overlapped with two genes (*PRAME* and *ZNF280B*, highlighted in bold in Table S9), which would result in misinterpretations about these regions. One CNVR on chr5 ranging from 58,386,640 to 58,441,130 contained six highly differentiated segments, whose loss type occurred in 66.33~79.93% of the Nellore cattle while it only happened in 4.75–6.32% of taurine cattle. We further examined this deletion region which may be under selection in Nellore cattle compared to taurine cattle (average *F*_*ST*_ = 0.43, Fig. 3b). The average LRRs were lower in Nellore cattle than that of taurine cattle and BAFs were dispersedly distributed in Nellore cattle. In taurine cattle, LRR and BAF values were clearly clustered as genotypes with two alleles, which implied the deletion may be a true positive event and was enriched in Nellore cattle. We found that five genes overlapped with the differential CNVRs. Since the cattle genome assembly and gene annotation were not as complete as human and rodents, especially for those diverse regions, we compared the sequence of each CNVRs across species in the UCSC genome browser (Table S9). Ortholog sequences of 12 CNVRs contained OR (olfactory receptor) related genes in other species and ortholog sequences of 4 other CNVRs contained ABC transporter related genes in other species. The sequence similar to OR and ABC transporter genes in cattle may be associated with the segregation of Nellore cattle from taurine breeds.

### Common CNVRs shared between Nellore and taurine cattle

We filtered away CNV segments with frequencies lower than 0.05 in the two populations and defined the left as common CNV segments, which were further merged into 96 CNVRs with 9.6 Mbp length. There were 25 genes in the RefGene database overlapped with the CNVRs (Table S10). We found three CNVRs with 0.59 Mbp overlapped with 4 genes that located in the false positive CNVRs. Using DAVID online software (http://david.abcc.ncifcrf.gov/), we annotated and classified ontologies for the 20 overlapped genes. One gene group including six immune related genes (*BOLA-DQA2*, *BOLA-DQA5*, *BOLA-DQB*, *BLA-DQB*, *BOLA* and *JSP.1*) was enriched with a score of 5.7. These six genes overlapped with 17 CNV segments which were merged into 5 CNVRs. All CNV segments overlapped with the MHC genes were located on chr23 from 25,350,763 to 28,494,392. The MHC genes were known to be highly polymorphic in humans, mice and other mammals^39^. In previous studies, genes related to adaptive immunity in CNVRs were found commonly shared in different species such as human, mouse, dog and cattle. Combining with the results of different CNVR coverage on chromosomes, our study provided more detailed evidences for the role of adaptive immunity genes in the population diversification. These multiple member gene families (e.g. olfactory receptor, ATP-binding cassette, and major histocompatibility complex genes) went through the so-called “birth-and-death” evolution^40^. In this model, they expand and contract – likely due to NAHR (non-allelic homologous recombination), FOSTES (fork stalling and template switching) or NHEJ (non-homologous end joining) mechanisms–and are subjected to either diversifying or stabilizing selection^41^.

## Discussion

The imperfect genome assembly, especially in the complex structure regions, influences the accuracy of CNV discovery. False positive CNVRs were also superficially studied in previous cattle CNV studies using the UMD3.1 reference assembly^42^,^43^,^44^. To our knowledge, this study is the first to report in detail that false positive CNVRs caused by misassemblies of the cattle genome through the comparison of the frequency of the CNV segments between female and male samples.

The UMD3.1 reference assembly lacked chrY information^45^ and some sequences from sex chromosomes were misplaced on autosomes. Additionally, repeat sequences took up 42~76% of the length of the nine false positive CNVRs, which indicated that there may be difficulty in assembling these regions. We used PennCNV in this study, which relies on SNP probe signal intensities to detect CNVs^37^. It’s easier to identify the sequence misassigned from chrY than from chrX to autosomes, because chrY is present only in males but not in females. According to the manufacturer’s SNP chip documentation, SNP logR ratios are adjusted separately for chrX and chrY. However, the regions misassigned from chrX or chrY to autosomes were not subject to these adjustments. As shown for chrX sequences in our study, logR ratios were slightly higher than 0 for female samples and slightly lower than 0 for male samples. The fluctuation of the signal intensity could lead to miscalling of CNVs. This might explain the differences of the CNV frequencies between Nellore and the HapMap cattle for false positive CNVs caused by misassignment of genomic regions from chrX to the autosomes (Fig. 2d).

**Figure 4:**
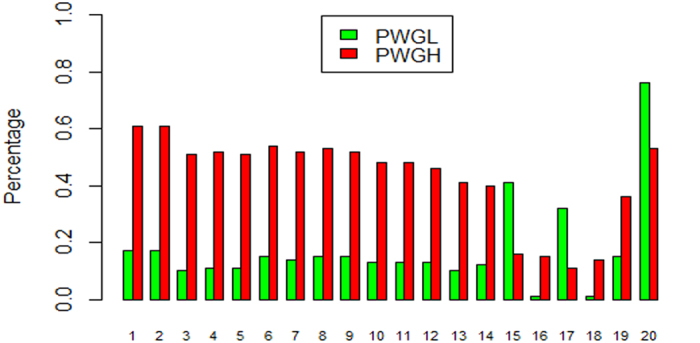
Effects of false positive CNVs on association study for PWG. Y-axis: Percentage of individuals with loss type; X-axis: Significantly associated CNV segments (p < 0.01). The first 14 CNV segments were located in false positive CNVRs.

As an important source of genetic variation, CNVs have been used in the association studies^43^. We separately selected 200 samples with extreme EBV values for both birth weight (BW) and post weaning gain (PWG) among the 1,682 Nellore cattle to evaluate the effects of false positive CNVRs on the results. The gender distributions in the two groups were almost even for BW and seriously unbalanced for PWG (Table S11). We merged the unique CNVs for the 200 samples and divided them to CNV segments and compared their loss variant frequencies using chi-square method. Even though no significant CNV segments were located in the false positive CNVRs for BW, 14 significantly differentiated CNV segments were found located in the false positive CNVRs for PWG. Most of the previously identified false positive CNVRs for PWG had the highest significance values (top 14 of the 20 significantly different segments in Fig. 4). Thus, false positive CNVRs should be carefully removed especially for the unbalanced male and female samples and gender linked traits, otherwise they will lead to incorrect results.

Using the unfiltered data, we compared the Nellore and taurine cattle at CNV level and evaluated the influence of false positive CNVs. Two false positive CNVRs comprising 0.13 Mbp and three false positive CNVRs comprising 0.59 Mbp appeared in the breed-differential CNVRs and high frequency CNVRs, respectively. Moreover, the overlapping result between the false positive CNVRs and the cattle QTL database showed 6 false positive CNVRs overlapped with 42 QTLs (Table 1). The QTLs overlapped in these wrongly assembled regions will seriously affect the working direction for the further studies as many researchers preferred reporting CNVs overlapped by QTLs.

By comparing the CNV frequency between Nellore cattle and taurine cattle, we found one 54-kb copy number deletion on chr5 significantly enriched in Nellore cattle. This region was consistent with one study which identified this region to be specific to *Bos indicus* cattle and associated with reproductive efficiency in *Bos indicus*-influenced cattle^46^. However, in our study, we did detect this deletion in taurine breeds with a low frequency. These may be related to the limited sample size used in the previous study. Deletions may cause difficulties in genotyping and imputing because of the lack of genomic DNA for hybridization. There were 26 QTLs with 12 traits were found overlapped with this high frequency deletion (Table S12). Eight of these traits were related to beef quality and quantity, including intramuscular fat, marbling score, longissimus muscle area, fat thickness at the 12^th^ rib and carcass weight. Genotyping errors may affect the study of beef quality QTLs in this region, especially for Nellore, which is bred primarily for beef. This deletion CNVR on chr5 should be carefully considered when doing the association study in Nellore cattle. For example, an Ensembl gene (*LOC782430*) coding for an OR protein was located in this region. Although this may support that this OR gene showed diversity between Nellore and taurine cattle, a conclusion attributing the QTL causes to this OR gene may be overreaching.

## Conclusion

In this study, we detected CNVs using a large number of Nellore cattle genotypes based on the BovineHD chip, which improved accuracy over previous studies because of the high density of SNP markers on the chip and more uniform coverage of these markers on chromosomes. By comparing our results with HapMap results, we constructed CNVR profiles for each chromosome. Considering the inconsistent frequencies among the different parts of one CNVR, we divided the CNVRs to CNV segments to do more detail studies. We then found and verified 9 false positive CNVRs comprising 0.8 Mb, which were caused by the misassembly of sequence from sex chromosomes to autosomes. We further evaluated the effects of false positive CNVRs on body traits study using two typical body traits (gender-unlinked: BW; gender-linked: PWG). We concluded that the false positive CNVRs should be carefully removed especially for the unbalanced male and female samples and gender-linked traits. Similar misassemblies are likely to be present in the reference genomes of other species. Targeted correcting them to produce more accurate assemblies will greatly increase the power of whole-genome resequencing and genome-wide association studies in these species^47^. Finally, we demonstrated that the lineage differentiated deletion CNV on chr5 should be carefully considered when doing the association study in Nellore cattle.

## Methods

### Samples

A total of 952 Nellore bulls and 1,278 Nellore cows were genotyped for 777,962 SNPs with the Illumina^®^ BovineHD Genotyping BeadChip assay, according to the manufacturer’s protocol (Illumina Inc.; 2011 BovineHD Genotyping BeadChip Data Sheet: DNA Analysis. http://www.illumina.com/Documents/products/datasheets/datasheet_bovineHD.pdf). This dataset builds on the data reported by previous studies, and comprises part of the genomic selection reference population from a commercial breeding program (DeltaGen) ran by an alliance of Nellore cattle breeders from Brazil. The present study was exempt of the local ethical committee evaluation as it did not involve any experiment on animals. Additionally, genomic DNA was extracted either from commercialized semen straws (bulls) or stored hair (cows) samples. The HapMap data was reported previously with the PennCNV option of “-test^35^”.

### Identification of cattle CNVs

The PennCNV algorithm (version 1.0.0) was used to detect cattle CNV among all the cattle autosomes in this study. ChrX and chrUn (a merged unplaced scaffolds) were excluded to avoid errors caused by mapping uncertainty. We generated both the signal intensity (log R Ratio, LRR) and allelic intensity (B allele frequency, BAF) ratio from Illumina GenomeStudio Genotyping Module v1.0 software given the default clustering file for each SNP. The PFB file was calculated based on the BAF of each marker in this population. The gcmodel file was generated by calculating the GC content of the 1 Mbp genomic region surrounding each marker (500 kb each side) and was used to adjust the genomic waves. We filtered the low quality samples with the default thresholds: standard deviation (STD) of LRR as 0.30, BAF drift as 0.01 and waviness factors as 0.05. CNVRs were determined by aggregating overlapping CNVs identified across all the samples and defined as three types (loss, both and gain) according to their composed CNV types (loss and gain).

### Gene Ontology and overlapping with QTL analysis

Gene content of cattle CNV regions was assessed using RefGene annotation file in UCSC database (http://hgdownload.soe.ucsc.edu/goldenPath/bosTau6/database/). QTL database was downloaded from animal QTL database (http://www.animalgenome.org/cgi-bin/QTLdb/index). The overlapped cases were detected using R script (version x64 3.0.3) and defined as at least one bp overlap. The protein IDs for each gene was used to quarry gene ontology terms using AgriGo with Fisher’s exact test (http://bioinfo.cau.edu.cn/agriGO/).

### CNV segment analysis

CNV segments were produced using R package (GenomicRanges) from unique CNVs. The boundaries of each segment were recovered to the position of corresponding probe position by R script for further convenient analysis. We defined the genotype of CNV segment for each individual according to the type of unique CNV it belonged to as no overlap among unique CNVs in one sample. The F-statistics (*F*_*ST*_) value was calculated according to the formulation in previous study^48^,^49^. *F*_*ST*_ = (H _t_− H_s_) / H_t_ ; H_t_ = 1 − P t_i_^2^; t_i_ = ((x_i_ · N_x_) + (y_i_ · N_y_)) / (N_x_ + N_y_); H_s_ =((1 − P_xi_^2^) · N_x_ + (1 − P_yi_^2^) · N_y_) / (N_x_ + N_y_), where x_i_ and y_i_ are the population frequencies of allelic CNV segment number i (i = A_0_, A_1_, A_2_, A_3_, A_4_ or >A_4_) in population X and Y, respectively, N_x_ and N_y_ denote the number of individuals in population X and Y, and t_i_ is a weighted average of x_i_ and y_i_.

### Validation using quantitative PCR and male-specific PCR

Primers were designed for qPCR validation using the NCBI Primer-BLAST webtool (http://www.ncbi.nlm.nih.gov/tools/primer-blast/index.cgi?LINK_LOC=BlastHome). Primer information can be seen in an additional file (Table S13). SYBR green chemistry used to do qPCR with triplicate reactions and 25 μl reaction volume for each. All reactions were amplified on a bioRad MyIQ thermocycler. BTF3 was chosen as a reference for all qPCR experiments. The 2^−ΔΔCT^ method was employed to analyze the qPCR result, using a common reference Nellore sample^19^. For male-specific PCR, we used the DNA samples of the sequenced Hereford cow L1 Dominette 01449 for female and its sire L1 Domino 99375 for male. Their DNA samples were used to assemble the cattle genome reference assemblies and their American Hereford Association registration numbers are 42190680 and 41170496, respectively. The male-specific PCR was performed in 25 μl reaction volume according to the Taq DNA polymerase manufacturer’s instruction (QIGEN, Taq PCR Master Mix Kit).The PCR regimen was as follows: initial denaturation for 5 min at 95 °C; followed by 36 cycles of 94 °C for 30 s, annealing at 60 °C for 25 s, and primer extension at 72 °C for 1 min; The final extension was performed at 72 °C for 10 min.

## Additional Information

**How to cite this article**: Zhou, Y. *et al*. Comparative analyses across cattle genders and breeds reveal the pitfalls caused by false positive and lineage-differential copy number variations. *Sci. Rep*. **6**, 29219; doi: 10.1038/srep29219 (2016).

## Supplementary Material

Supplementary Information

Supplementary Table S1-S4

Supplementary Table S5-S13

## Figures and Tables

**Table 1:** False positive CNVRs caused by genome assembly errors.

False positive CNVRs	UMD3.1Chr	Start	End	Btau_4.6.1Chr	Overlapped gene	Overlapped with QTLs
FP_CNVR1	chr1	83,218,713	83,238,102	chrY	*EPHB3*	Body weight (yearling)
FP_CNVR2	chr2	55,587,169	55,598,367	chrUn		
FP_CNVR3	chr4	45,054,839	45,072,215	chrX	*RELN*	Stillbirth (direct), Calving ease (maternal)
FP_CNVR4	chr4	75,028,395	75,104,623	chrUn	*GTPBP10*	
FP_CNVR5	chr5	22,514,133	22,563,988	chrY		Intramuscular fat, Shear force, Longissimus muscle area, Fat thickness at the 12th rib, Kidney fat weight, Carcass weight, Body weight (slaughter), Chest width, Length of productive life, Milk fat percentage, Milk fat yield (daughter deviation), Body condition score, Somatic cell score (DYD), Conformation score, Rump angle
FP_CNVR6	chr17	15,448,739	15,477,125	chrX		Marbling score
FP_CNVR7	chr17	25,056,695	25,119,996	chrY	*PRAME*	
FP_CNVR8	chr17	50,746,686	50,962,760	chrY		Trans-16-C18:1 fatty acid content, Clinical mastitis (DYD), Shear force, Somatic cell score (DYD)
FP_CNVR9	chr17	51,115,979	51,433,906	chrY	*ZNF280B*, *HFSY2*, *PRAME*	Trans-16-C18:1 fatty acid content, Clinical mastitis (DYD), Shear force, Metabolic body weight, Dry matter intake, Abomasum displacement, Body weight (weaning)
