# Supplementary material for: Comparative analyses across cattle genders and breeds reveal the pitfalls caused by false positive and lineage-differential copy number variations
Source: Sci Rep. 2016 Jul 6;6:29219. doi: 10.1038/srep29219 (PMC4933914; doi:10.1038/srep29219)
Supplement: Supplementary Information [file srep29219-s1.pdf]

Comparative analyses across cattle breeds reveal the pitfalls caused by false positive and lineage-differential copy number variations

Yang Zhou[1,2], Yuri T. Utsunomiya[3], Lingyang Xu[1,4], El Hamidi abdel Hay[1], Derek M. Bickhart[1], Tad S. Sonstegard[1, §], Curtis P. Van Tassell[1], Jose Fernando Garcia[3,5,†] and George E. Liu[1,†]

Supplementary Information

Supplementary file 1: Table S1 to S4;
Supplementary file 2: Table S5 to S13.

Supplementary Figures

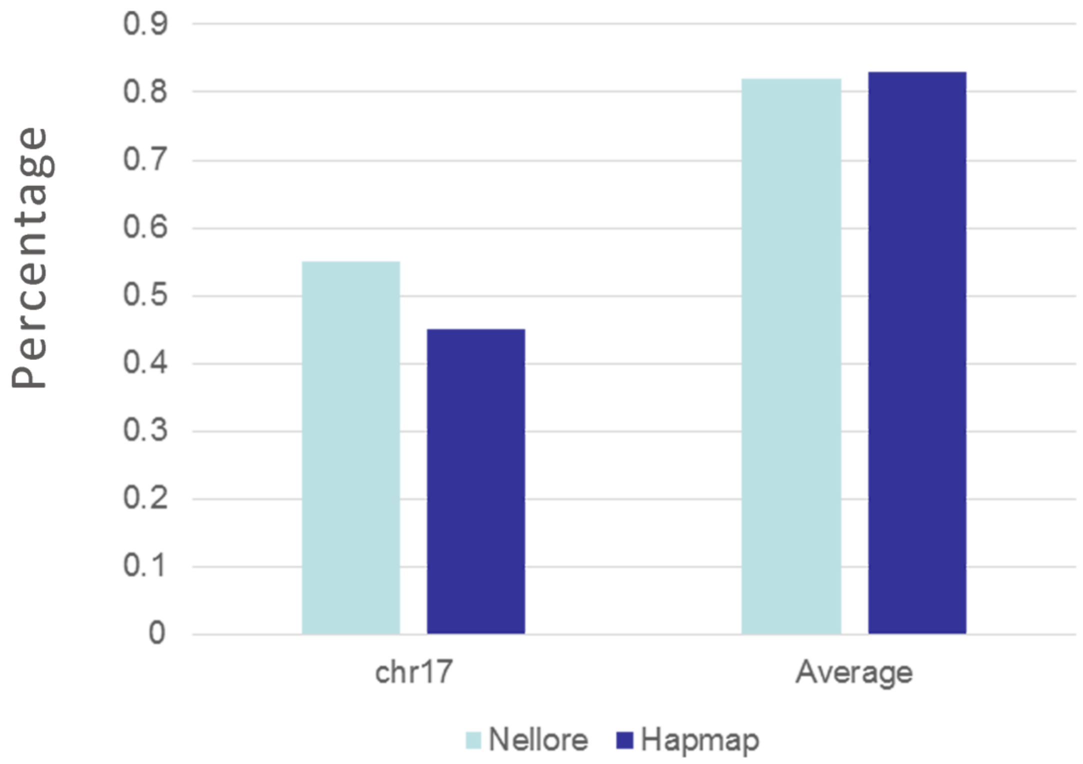

Figure S1. Chr17 has shorter overlap regions among CNVs than the average of autosomes.

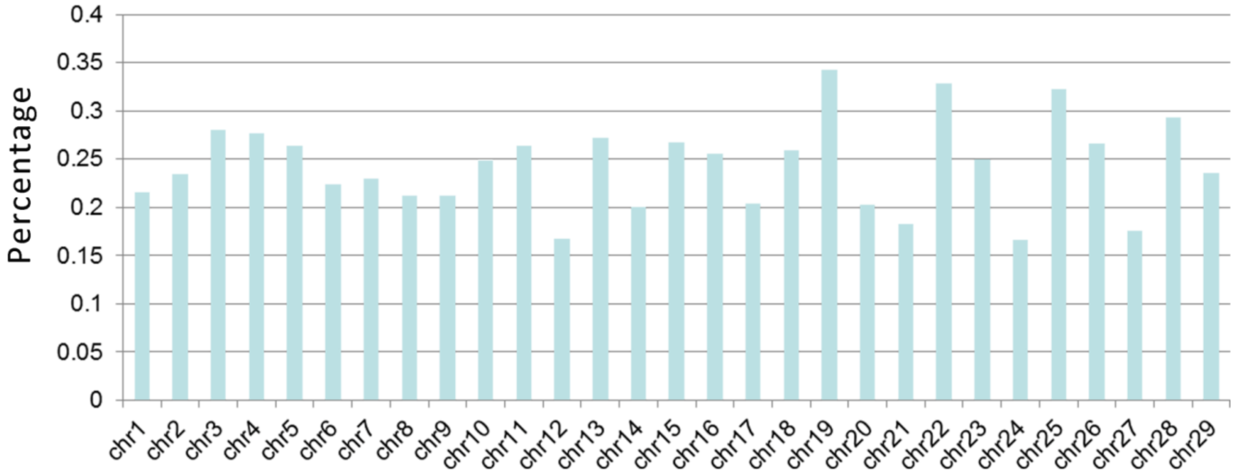

Figure S2. Percentage of genic region on each autosome.

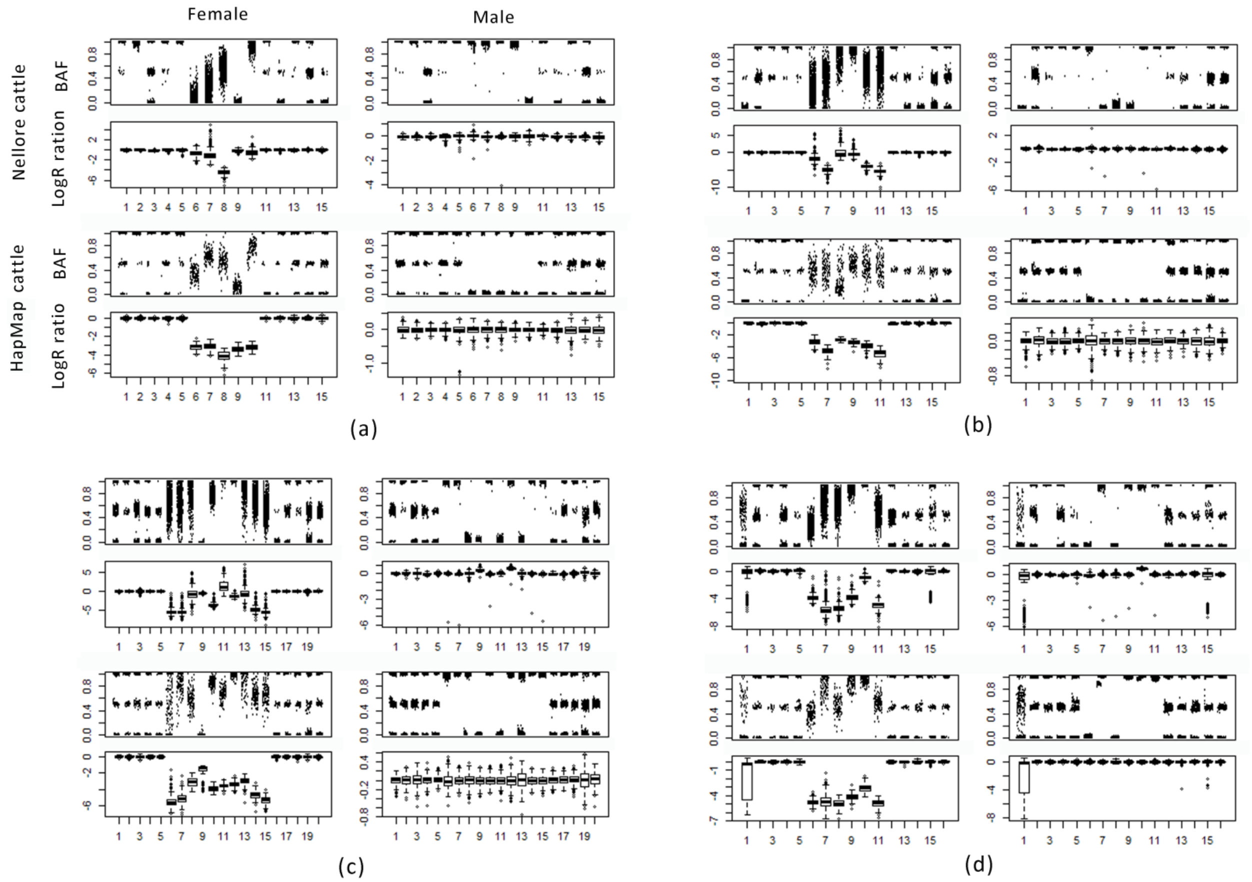

Figure S3. B allele frequency distribution and Log R ratio values for SNP probes near false positive CNVRs caused by assembly errors of sequences from chrY. Five upstream and downstream probes were included to represent the basal level. (a) FP_CNVR1; (b) FP_CNVR5; (c) FP_CNVR8; and (d) FP_CNVR9.

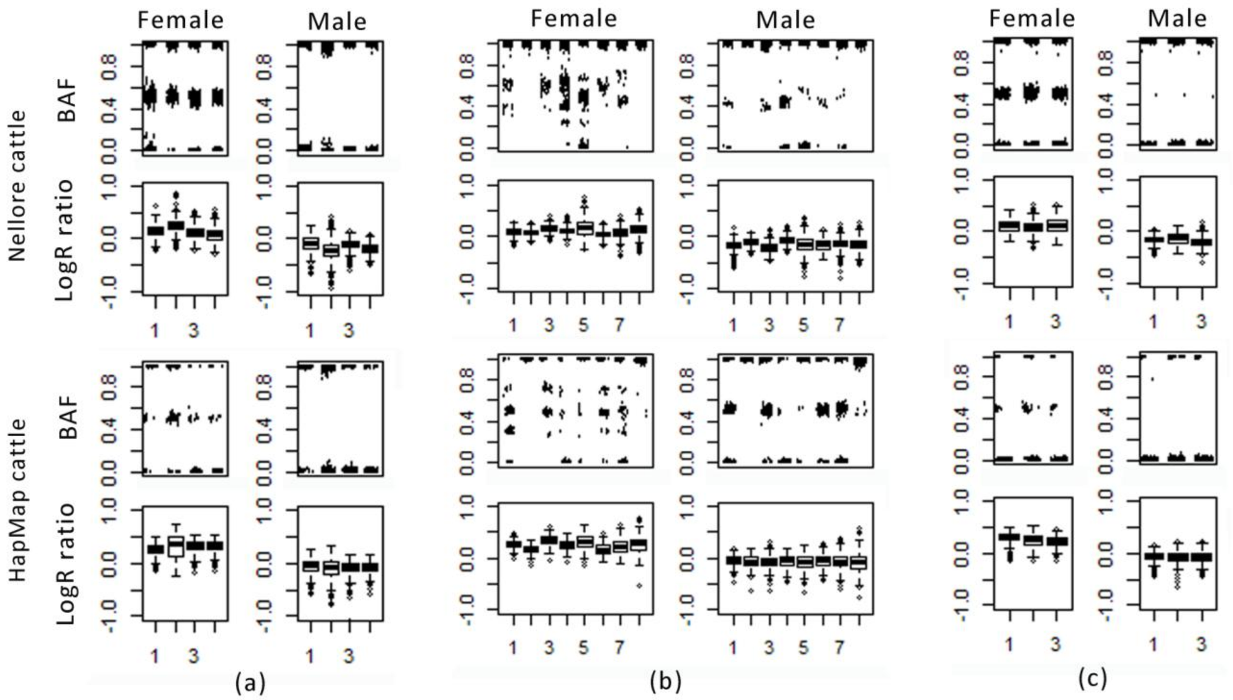

Figure S4. B allele frequency distribution and LogR ratio values for SNP probes in false positive CNVRs caused by assembly errors of sequences from chrX and ChrUn. (a) FP_CNVR3; (b) FP_CNVR4; and (c) FP_CNVR6.
